# 7,7′,8,8′-Tetra­meth­oxy-4,4′-dimethyl-3,3′-bicoumarin

**DOI:** 10.1107/S1600536809017334

**Published:** 2009-05-14

**Authors:** Hoong-Kun Fun, Samuel Robinson Jebas, Mehtab Parveen, Zakia Khanam, Raza Murad Ghalib

**Affiliations:** a X-ray Crystallography Unit, School of Physics, Universiti Sains Malaysia, 11800 Universiti Sains Malaysia, Penang, Malaysia; b Department of Chemistry, Aligarh Muslim University, Aligarh 202002 (UP), India

## Abstract

In the crystal structure, the title compound, C_24_H_22_O_8_, lies on a twofold rotation axis and the asymmetric unit comprises one half-mol­ecule. The dihedral angle formed by the coumarin unit with the symmetry-related part is 74.78 (14)°. One of the meth­oxy groups attached to the coumarin unit is considerably twisted, making an angle of 87.17 (17)° with respect to the coumarin unit; the other is twisted by 0.66 (19)°. No classical hydrogen bonds are found in the sturcture; only a weak C—H⋯π inter­action and short intra­molecular O⋯O contacts [2.683 (2)–2.701 (2) Å] are observed.

## Related literature

For the biological activity of coumarins, see: El-Agrody *et al.* (2001 ▶); El-Farargy (1991 ▶); Emmanuel-Giota *et al.* (2001 ▶); Ghate *et al.* (2005 ▶); Laakso *et al.* (1994 ▶); Nofal *et al.* (2000 ▶); Pratibha & Shreeya (1999 ▶); Shaker (1996 ▶); Yang *et al.* (2005 ▶). For the pharmaceutical properties of coumarin derivatives, see: Kennedy & Thornes (1997 ▶). For natural and synthetic coumarins, see: Carlton *et al.* (1996 ▶); Zhou *et al.* (2000 ▶). For related bond-length data, see: Allen *et al.* (1987 ▶). For stability of the temperature controller used in the data collection, see: Cosier & Glazer (1986 ▶).
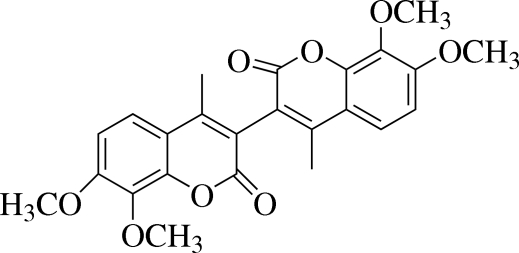


## Experimental

### 

#### Crystal data

C_24_H_22_O_8_
*M* *_r_* = 438.42
Monoclinic, 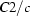

*a* = 21.715 (9) Å
*b* = 7.138 (3) Å
*c* = 15.511 (6) Å
β = 121.801 (5)°
*V* = 2043.3 (14) Å^3^
*Z* = 4
Mo *K*α radiation
μ = 0.11 mm^−1^
*T* = 100 K
0.28 × 0.19 × 0.06 mm

#### Data collection

Bruker SMART APEXII CCD area-detector diffractometer
Absorption correction: multi-scan (**SADABS**; Bruker, 2005 ▶) *T*_min_ = 0.971, *T*_max_ = 0.994
27961 measured reflections
3527 independent reflections
2710 reflections with *I* > 2σ(*I*)
*R*_int_ = 0.065

#### Refinement

*R*[*F* ^2^ > 2σ(*F* ^2^)] = 0.059
*wR*(*F* ^2^) = 0.155
*S* = 1.08
3527 reflections
189 parameters
All H-atom parameters refined
Δρ_max_ = 0.50 e Å^−3^
Δρ_min_ = −0.21 e Å^−3^

### 

Data collection: *APEX2* (Bruker, 2005 ▶); cell refinement: *SAINT* (Bruker, 2005 ▶); data reduction: *SAINT*; program(s) used to solve structure: *SHELXTL* (Sheldrick, 2008 ▶); program(s) used to refine structure: *SHELXTL*; molecular graphics: *SHELXTL*; software used to prepare material for publication: *SHELXTL* and *PLATON* (Spek, 2009 ▶).

## Supplementary Material

Crystal structure: contains datablocks global, I. DOI: 10.1107/S1600536809017334/is2417sup1.cif

Structure factors: contains datablocks I. DOI: 10.1107/S1600536809017334/is2417Isup2.hkl

Additional supplementary materials:  crystallographic information; 3D view; checkCIF report

## Figures and Tables

**Table 1:** Hydrogen-bond geometry (Å, °)

*D*—H⋯*A*	*D*—H	H⋯*A*	*D*⋯*A*	*D*—H⋯*A*
C6—H6⋯*Cg*1^i^	0.96 (2)	2.86 (2)	3.676 (2)	143.5 (18)

Symmetry code: (i) 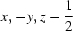. *Cg*1 is the centroid of the C4–C9 ring.

**Fig. 1.**
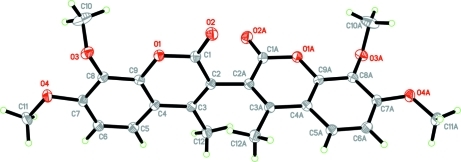
The molecular structure of (I), showing 50% probability displacement ellipsoids and the atom numbering scheme. [Symmetry code: -x, y, -z + 1/2 to generate equivalent atoms].

**Fig. 2.**
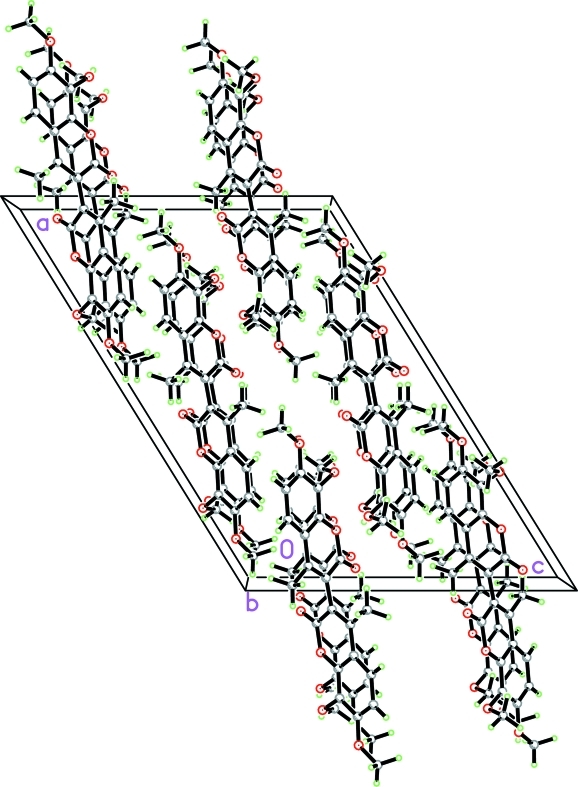
The crystal packing of (I). Molecules are stacked along the b axis.

## supplementary crystallographic information

### Comment

Coumarins are a large group of naturally occurring oxygen heterocycles representing 2*H*-1-benzopyran-2-one derivative. Many natural coumarins are reputed for their wide range of biological activites such as antibacterial (El-Agrody *et al.*, 2001; Pratibha & Shreeya, 1999), antifungal (Shaker, 1996; El-Farargy, 1991), antioxidant (Yang *et al.*, 2005), analgesic (Ghate *et al.*, 2005), anti-inflammatory (Emmanuel-Giota *et al.*, 2001) and antitumor (Nofal *et al.*, 2000) properties. Bi and tri-coumarins are comparatively new groups which are widely spread in nature and their biological properties are also well known (Laakso *et al.*, 1994). One of the characteristic pharmacological properties of coumarin derivatives is the anticoagulant action (Kennedy & Thornes, 1997). A large number of natural and semisynthetic coumarin and bicoumarin derivatives have been reported to demonstrate chemopreventive (Carlton *et al.*, 1996) and anti-HIV (Zhou *et al.*, 2000) activities. Keeping in view of these biological importance of coumarins and their dimers, we have synthesized the title compound (I) and report here its structure.

The asymmetric unit of (I) (Fig. 1), contains half of the 7,7',8,8'-4,4'-dimethyl-3,3'-bicoumarin molecule. The other half is symmetry generated [symmetry code: -*x*, *y*, -*z* + 1/2]. The coumarin unit is planar with the maximum deviation from the mean plane of 0.0295 (15) Å for atom C2. One of the methyl group attached to the coumarin unit is twisted as evidenced by the torsion angle of C10—O3—C8—C9 = 87.17 (17)°. The dihedral angle formed by the coumarin unit (O1/C1—C9) with the symmetry related coumarin unit (O1A/C1A—C9A) is 74.78 (14)°, indicating that they are almost perpendicular to each other. The bond lengths (Allen *et al.*, 1987) and bond angles are normal.

The crystal packing (Fig. 2) (Table 1) is stabilized by weak C—H···π interactions and intramolecular O···O = 2.683 (2) to 2.701 (2) Å short contacts.

### Experimental

A mixture of 7,8-dimethoxy-4-methyl coumarin (2.20 g, 10 mmol) and manganese(III) acetate (0.774 g, 1 mmol) was stirred at room temperature, then 70% perchloric acid (0.8 g, 6 mmol) was added. The reaction mixture was heated under reflux at 114°C with stirring in the atmosphere of nitrogen for 3 h. The reaction mixture was cooled and diluted with 50 ml of benzene. The benzene solution was washed with water and aq. NaHCO_3_, dried over anhydrous Na_2_SO_4_ and left to evaporate. The residue showed two major compounds which were separated by column chromatography followed by preparative thin layer chromatography (Benzene: EtOAc, 9:1) into the title compound (I) (260 mg, 12%).

### Refinement

All the hydrogen atoms were located from the Fourier map and allowed to refine freely.

### Crystal data

**Table:**

C_24_H_22_O_8_	*F*(000) = 920
*M**_r_* = 438.42	*D*_x_ = 1.425 Mg m^−^^3^
Monoclinic, *C*2/*c*	Mo *K*α radiation, λ = 0.71073 Å
Hall symbol: -C 2yc	Cell parameters from 6562 reflections
*a* = 21.715 (9) Å	θ = 2.7–31.8°
*b* = 7.138 (3) Å	µ = 0.11 mm^−^^1^
*c* = 15.511 (6) Å	*T* = 100 K
β = 121.801 (5)°	Plate, colourless
*V* = 2043.3 (14) Å^3^	0.28 × 0.19 × 0.06 mm
*Z* = 4	

### Data collection

**Table:**

Bruker SMART APEXII CCD area-detector diffractometer	3527 independent reflections
Radiation source: fine-focus sealed tube	2710 reflections with *I* > 2σ(*I*)
graphite	*R*_int_ = 0.065
φ and ω scans	θ_max_ = 32.0°, θ_min_ = 2.2°
Absorption correction: multi-scan (*SADABS*; Bruker, 2005)	*h* = −32→32
*T*_min_ = 0.971, *T*_max_ = 0.994	*k* = −10→10
27961 measured reflections	*l* = −23→23

### Refinement

**Table:**

Refinement on *F*^2^	Primary atom site location: structure-invariant direct methods
Least-squares matrix: full	Secondary atom site location: difference Fourier map
*R*[*F*^2^ > 2σ(*F*^2^)] = 0.059	Hydrogen site location: inferred from neighbouring sites
*wR*(*F*^2^) = 0.155	All H-atom parameters refined
*S* = 1.08	*w* = 1/[σ^2^(*F*_o_^2^) + (0.0752*P*)^2^ + 1.2567*P*] where *P* = (*F*_o_^2^ + 2*F*_c_^2^)/3
3527 reflections	(Δ/σ)_max_ < 0.001
189 parameters	Δρ_max_ = 0.50 e Å^−^^3^
0 restraints	Δρ_min_ = −0.21 e Å^−^^3^

### Special details

**Table:**

Experimental. The crystal was placed in the cold stream of an Oxford Cyrosystems Cobra open-flow nitrogen cryostat (Cosier & Glazer, 1986) operating at 100.0 (1) K.
Geometry. All e.s.d.'s (except the e.s.d. in the dihedral angle between two l.s. planes) are estimated using the full covariance matrix. The cell e.s.d.'s are taken into account individually in the estimation of e.s.d.'s in distances, angles and torsion angles; correlations between e.s.d.'s in cell parameters are only used when they are defined by crystal symmetry. An approximate (isotropic) treatment of cell e.s.d.'s is used for estimating e.s.d.'s involving l.s. planes.
Refinement. Refinement of *F*^2^ against ALL reflections. The weighted *R*-factor *wR* and goodness of fit *S* are based on *F*^2^, conventional *R*-factors *R* are based on *F*, with *F* set to zero for negative *F*^2^. The threshold expression of *F*^2^ > σ(*F*^2^) is used only for calculating *R*-factors(gt) *etc*. and is not relevant to the choice of reflections for refinement. *R*-factors based on *F*^2^ are statistically about twice as large as those based on *F*, and *R*- factors based on ALL data will be even larger.

### Fractional atomic coordinates and isotropic or equivalent isotropic displacement parameters (Å^2^)

**Table:**

	*x*	*y*	*z*	*U*_iso_*/*U*_eq_	
O1	0.15443 (5)	0.04445 (13)	0.38705 (7)	0.0178 (2)	
O2	0.05473 (6)	−0.06775 (15)	0.37300 (8)	0.0242 (2)	
O3	0.30066 (5)	0.03278 (14)	0.48961 (7)	0.0212 (2)	
O4	0.37511 (5)	0.26053 (15)	0.43926 (8)	0.0221 (2)	
C1	0.08045 (7)	0.05014 (19)	0.34517 (10)	0.0175 (3)	
C2	0.03949 (7)	0.19639 (19)	0.27048 (10)	0.0163 (3)	
C3	0.07228 (7)	0.32194 (19)	0.24158 (10)	0.0168 (3)	
C4	0.15015 (7)	0.31354 (18)	0.28870 (10)	0.0161 (3)	
C5	0.19034 (8)	0.44106 (19)	0.26927 (10)	0.0185 (3)	
C6	0.26498 (8)	0.42852 (19)	0.31768 (11)	0.0194 (3)	
C7	0.30199 (7)	0.28719 (19)	0.38881 (10)	0.0174 (3)	
C8	0.26401 (7)	0.15966 (18)	0.41265 (9)	0.0164 (3)	
C9	0.18892 (7)	0.17416 (18)	0.36139 (10)	0.0154 (2)	
C10	0.30773 (13)	−0.1493 (2)	0.45770 (15)	0.0362 (4)	
C11	0.41539 (8)	0.3869 (2)	0.41549 (13)	0.0267 (3)	
C12	0.02974 (8)	0.4697 (2)	0.16315 (12)	0.0247 (3)	
H5	0.1654 (10)	0.540 (3)	0.2226 (15)	0.024 (5)*	
H6	0.2908 (11)	0.516 (3)	0.3016 (16)	0.033 (5)*	
H10A	0.3396 (14)	−0.139 (4)	0.429 (2)	0.063 (8)*	
H10B	0.3324 (13)	−0.224 (3)	0.514 (2)	0.046 (6)*	
H10C	0.2573 (16)	−0.202 (4)	0.410 (2)	0.067 (8)*	
H11A	0.3985 (12)	0.382 (3)	0.3405 (19)	0.044 (6)*	
H11B	0.4125 (11)	0.519 (3)	0.4338 (15)	0.028 (5)*	
H11C	0.4642 (10)	0.340 (3)	0.4535 (14)	0.022 (4)*	
H12A	0.0472 (11)	0.483 (3)	0.1149 (17)	0.035 (5)*	
H12B	0.0359 (12)	0.591 (3)	0.1955 (18)	0.041 (6)*	
H12C	−0.0217 (12)	0.440 (3)	0.1264 (17)	0.036 (5)*	

### Atomic displacement parameters (Å^2^)

**Table:**

	*U*^11^	*U*^22^	*U*^33^	*U*^12^	*U*^13^	*U*^23^
O1	0.0162 (5)	0.0193 (5)	0.0163 (4)	0.0013 (3)	0.0074 (4)	0.0050 (4)
O2	0.0213 (5)	0.0253 (5)	0.0244 (5)	−0.0003 (4)	0.0111 (4)	0.0083 (4)
O3	0.0217 (5)	0.0219 (5)	0.0134 (4)	0.0047 (4)	0.0048 (4)	0.0029 (4)
O4	0.0144 (5)	0.0250 (5)	0.0230 (5)	−0.0015 (4)	0.0071 (4)	−0.0020 (4)
C1	0.0167 (6)	0.0199 (6)	0.0149 (6)	0.0003 (5)	0.0076 (5)	0.0008 (5)
C2	0.0155 (6)	0.0179 (6)	0.0140 (6)	0.0004 (4)	0.0067 (5)	−0.0003 (4)
C3	0.0166 (6)	0.0176 (6)	0.0145 (6)	0.0013 (4)	0.0071 (5)	0.0018 (4)
C4	0.0166 (6)	0.0174 (6)	0.0133 (5)	0.0013 (4)	0.0072 (5)	0.0009 (4)
C5	0.0199 (6)	0.0185 (6)	0.0165 (6)	0.0007 (5)	0.0091 (5)	0.0027 (5)
C6	0.0202 (6)	0.0200 (6)	0.0186 (6)	−0.0016 (5)	0.0106 (5)	−0.0002 (5)
C7	0.0142 (6)	0.0211 (6)	0.0143 (6)	−0.0007 (4)	0.0058 (5)	−0.0037 (5)
C8	0.0168 (6)	0.0176 (6)	0.0108 (5)	0.0018 (4)	0.0045 (5)	−0.0003 (4)
C9	0.0175 (6)	0.0155 (6)	0.0125 (5)	−0.0012 (4)	0.0075 (5)	−0.0006 (4)
C10	0.0564 (12)	0.0239 (8)	0.0302 (9)	0.0173 (8)	0.0241 (9)	0.0093 (7)
C11	0.0188 (7)	0.0267 (8)	0.0339 (8)	−0.0059 (6)	0.0134 (6)	−0.0047 (6)
C12	0.0183 (7)	0.0265 (7)	0.0254 (7)	0.0030 (5)	0.0089 (6)	0.0112 (6)

### Geometric parameters (Å, °)

**Table:**

O1—C9	1.3750 (16)	C5—H5	0.952 (19)
O1—C1	1.3801 (17)	C6—C7	1.395 (2)
O2—C1	1.2085 (17)	C6—H6	0.96 (2)
O3—C8	1.3701 (16)	C7—C8	1.4025 (19)
O3—C10	1.428 (2)	C8—C9	1.3912 (19)
O4—C7	1.3640 (17)	C10—H10A	1.01 (3)
O4—C11	1.4334 (19)	C10—H10B	0.92 (3)
C1—C2	1.4618 (19)	C10—H10C	1.02 (3)
C2—C3	1.3592 (19)	C11—H11A	1.02 (2)
C2—C2^i^	1.482 (3)	C11—H11B	1.00 (2)
C3—C4	1.4472 (19)	C11—H11C	0.963 (19)
C3—C12	1.5036 (19)	C12—H12A	1.01 (2)
C4—C5	1.3993 (19)	C12—H12B	0.97 (2)
C4—C9	1.4034 (18)	C12—H12C	0.97 (2)
C5—C6	1.384 (2)		
			
C9—O1—C1	121.36 (10)	O3—C8—C9	121.00 (12)
C8—O3—C10	114.77 (12)	O3—C8—C7	120.42 (12)
C7—O4—C11	116.63 (12)	C9—C8—C7	118.42 (12)
O2—C1—O1	116.99 (12)	O1—C9—C8	115.95 (11)
O2—C1—C2	125.28 (13)	O1—C9—C4	121.49 (12)
O1—C1—C2	117.72 (11)	C8—C9—C4	122.56 (12)
C3—C2—C1	121.77 (12)	O3—C10—H10A	108.3 (16)
C3—C2—C2^i^	123.20 (11)	O3—C10—H10B	108.3 (15)
C1—C2—C2^i^	115.03 (10)	H10A—C10—H10B	106 (2)
C2—C3—C4	118.80 (12)	O3—C10—H10C	108.6 (16)
C2—C3—C12	121.60 (13)	H10A—C10—H10C	115 (2)
C4—C3—C12	119.59 (12)	H10B—C10—H10C	110 (2)
C5—C4—C9	117.13 (12)	O4—C11—H11A	111.7 (13)
C5—C4—C3	124.03 (12)	O4—C11—H11B	112.6 (11)
C9—C4—C3	118.80 (12)	H11A—C11—H11B	108.8 (17)
C6—C5—C4	121.74 (13)	O4—C11—H11C	104.2 (11)
C6—C5—H5	119.7 (11)	H11A—C11—H11C	107.7 (16)
C4—C5—H5	118.6 (11)	H11B—C11—H11C	111.8 (16)
C5—C6—C7	119.83 (13)	C3—C12—H12A	111.0 (12)
C5—C6—H6	119.7 (13)	C3—C12—H12B	110.3 (14)
C7—C6—H6	120.5 (13)	H12A—C12—H12B	107.2 (17)
O4—C7—C6	124.46 (12)	C3—C12—H12C	110.3 (12)
O4—C7—C8	115.26 (12)	H12A—C12—H12C	110.5 (18)
C6—C7—C8	120.28 (13)	H12B—C12—H12C	107.4 (17)
			
C9—O1—C1—O2	−179.03 (12)	C5—C6—C7—O4	178.75 (12)
C9—O1—C1—C2	1.45 (18)	C5—C6—C7—C8	−1.0 (2)
O2—C1—C2—C3	−178.65 (14)	C10—O3—C8—C9	87.17 (17)
O1—C1—C2—C3	0.83 (19)	C10—O3—C8—C7	−97.47 (17)
O2—C1—C2—C2^i^	1.3 (2)	O4—C7—C8—O3	6.82 (18)
O1—C1—C2—C2^i^	−179.20 (11)	C6—C7—C8—O3	−173.39 (12)
C1—C2—C3—C4	−2.0 (2)	O4—C7—C8—C9	−177.70 (11)
C2^i^—C2—C3—C4	178.01 (13)	C6—C7—C8—C9	2.09 (19)
C1—C2—C3—C12	178.72 (13)	C1—O1—C9—C8	176.73 (11)
C2^i^—C2—C3—C12	−1.3 (2)	C1—O1—C9—C4	−2.48 (18)
C2—C3—C4—C5	−176.43 (13)	O3—C8—C9—O1	−5.18 (18)
C12—C3—C4—C5	2.8 (2)	C7—C8—C9—O1	179.37 (11)
C2—C3—C4—C9	1.01 (19)	O3—C8—C9—C4	174.02 (12)
C12—C3—C4—C9	−179.71 (13)	C7—C8—C9—C4	−1.42 (19)
C9—C4—C5—C6	1.4 (2)	C5—C4—C9—O1	178.84 (11)
C3—C4—C5—C6	178.94 (13)	C3—C4—C9—O1	1.22 (19)
C4—C5—C6—C7	−0.8 (2)	C5—C4—C9—C8	−0.32 (19)
C11—O4—C7—C6	−0.66 (19)	C3—C4—C9—C8	−177.94 (12)
C11—O4—C7—C8	179.12 (12)		

Symmetry codes: (i) −*x*, *y*, −*z*+1/2.

### Hydrogen-bond geometry (Å, °)

**Table:**

*D*—H···*A*	*D*—H	H···*A*	*D*···*A*	*D*—H···*A*
C6—H6···Cg1^ii^	0.96 (2)	2.86 (2)	3.676 (2)	143.5 (18)

Symmetry codes: (ii) *x*, −*y*, *z*−1/2.
